# Association of Postoperative Stress Hyperglycemia Ratio with Suspected Infection, Culture Positivity, Sepsis, and Septic Shock in 1408 Patients After Cranial Neurosurgery

**DOI:** 10.3390/jcm15197505

**Published:** 2026-09-27

**Authors:** Dong Tang, Tianxiang Huang

**Affiliations:** 1 Department of Neurosurgery, Xiangya Hospital, Central South University, 87 Xiangya Road, Changsha 410008, China; 248102128@csu.edu.cn; 2 National Clinical Research Center for Geriatric Disorders, Xiangya Hospital, Central South University, 87 Xiangya Road, Changsha 410008, China; 3 Cerebrovascular Diseases Research Center, Xiangya Hospital, Central South University, 87 Xiangya Road, Changsha 410008, China

**Keywords:** stress hyperglycemia ratio, cranial neurosurgery, infection-related outcomes, Sepsis-3, septic shock, MIMIC-IV

## Abstract

**Background/Objectives:** The early postoperative stress hyperglycemia ratio (SHR) may capture acute glycemic stress relative to chronic glycemia, but its association with infection-related events after cranial neurosurgery remains uncertain. We evaluated these associations in critically ill patients after cranial neurosurgical procedures. **Methods:** We retrospectively analyzed 1408 adults in MIMIC-IV after eligible cranial neurosurgical procedures. SHR was calculated from the first serum glucose measurement 6–24 h after surgery and estimated average glucose was derived from HbA1c. Primary outcomes were electronic health record (EHR)-defined suspected infection, culture-positive infection from any qualifying specimen, Sepsis-3-defined sepsis, and an EHR-based septic shock proxy. The secondary outcome was 90-day mortality. **Results:** In fully adjusted models, each 0.1-unit increase in SHR was associated with suspected infection (OR 1.08, 95% CI 1.04–1.12), culture-positive infection (OR 1.05, 95% CI 1.01–1.09), Sepsis-3-defined sepsis (OR 1.07, 95% CI 1.03–1.11), and septic shock (OR 1.10, 95% CI 1.05–1.15). In nested analyses, each 1-SD increase in SHR was associated with 90-day mortality (OR 1.59, 95% CI 1.29–1.96). Associations persisted in 24-h lag analyses. Adding SHR yielded small, non-significant AUC increases but improved reclassification for Sepsis-3-defined sepsis and septic shock. **Conclusions:** Higher early postoperative SHR was associated with EHR-derived, all-source infection-related and sepsis-related events and 90-day mortality in a heterogeneous postoperative neurocritical care cohort. These findings do not establish that SHR predicts postoperative neurosurgical infection; prospective external validation is required before clinical use.

## 1. Introduction

Postoperative infection is among the most serious complications after craniotomy, particularly in patients who require postoperative intensive care. In neurosurgical populations, the clinical impact of infection is amplified by procedure-specific vulnerabilities, including prolonged operative duration, repeated cranial intervention, cerebrospinal fluid (CSF) leakage, ventricular or CSF drainage devices, postoperative intracranial complications, and the frequent need for invasive monitoring and organ support [1,2,3,4,5,6]. A recent systematic review of cranial surgery for brain tumors reported pooled surgical-site infection rates of 4.03% at 30 days and 6.17% at 90 days, with higher rates after posterior fossa procedures [1]. In a large single-center series, post-craniotomy intracranial infection occurred in 6.8% of procedures, and most cases were diagnosed within the first 2 postoperative weeks [2]. Across studies, CSF leakage, external CSF drainage, reoperation, prolonged operative time, greater preoperative vulnerability, and more urgent or complex procedures have been associated with infection risk [3,4]. Once infection occurs, the clinical consequences may include prolonged hospitalization, reoperation, neurological disability, organ dysfunction, and death [4,5,6]. In a prospective cohort of critically ill post-craniotomy patients, hospital mortality was 13.7% in patients with sepsis versus 8.3% in those without sepsis [5]. In a separate prospective neurocritical care cohort of patients with subarachnoid hemorrhage, long-term poor functional outcomes (modified Rankin Scale score 4–6) occurred in 66% of septic versus 21% of nonseptic patients, and mortality was 52.5% versus 16%, respectively [7]. These estimates vary with the underlying diagnosis, infection definition, and disease severity. These observations highlight the need for early postoperative markers that can help identify patients at increased risk of infection-related complications without assuming that all patients follow a single, uniform disease trajectory.

Stress hyperglycemia is a common metabolic response to major surgery and critical illness, driven by neuroendocrine and inflammatory pathways that increase insulin resistance and hepatic glucose production and alter peripheral glucose utilization [8]. SHR relates acute glucose to the estimated average glucose derived from glycated hemoglobin (HbA1c), thereby accounting for chronic glycemic background [9]. This distinction is relevant in mixed ICU populations with diabetes, prediabetes, and previously unrecognized dysglycemia. Acute stress hyperglycemia may promote infection and organ dysfunction by impairing neutrophil chemotaxis, phagocytosis, oxidative burst, microbial killing, and complement activity and by disrupting inflammatory signaling, endothelial function, and microvascular reactivity [10,11]. Pre-existing diabetes may add chronic immune dysfunction, advanced glycation, oxidative stress, microangiopathy, impaired tissue repair, and cerebral microvascular and blood–brain barrier injury [12,13]. The observed complications may therefore reflect both acute metabolic stress and chronic diabetes-related susceptibility. SHR cannot fully separate these contributions and may also mark surgical stress, tissue injury, or occult infection rather than act as a direct cause.

Higher SHR has been associated with adverse outcomes in acute ischemic stroke, cardiovascular disease, heterogeneous critical illness, and established sepsis [14,15,16,17,18]. Most sepsis studies have focused on mortality after sepsis is already present, whereas evidence relating relative hyperglycemia to earlier postoperative infection-related events is limited. In orthopedic trauma cohorts, stress-induced hyperglycemia has been associated with surgical-site infection [19,20], and in elective craniotomy, elevated preoperative HbA1c and severe intraoperative hyperglycemia have been associated with early postoperative infection [21]. However, these studies do not establish whether an early postoperative measure that accounts for chronic glycemia is associated with distinct infection-related outcomes in critically ill patients after cranial neurosurgery. Moreover, culture positivity, suspected infection, Sepsis-3-defined sepsis, and septic shock capture different clinical constructs. Culture results depend on specimen type, timing, prior antimicrobial exposure, contamination, and local testing practice, whereas Sepsis-3-defined sepsis and septic shock additionally reflect organ dysfunction and circulatory or metabolic abnormalities [22]. Evaluating these outcomes separately may therefore provide a more clinically informative assessment than treating postoperative infection as a single binary endpoint.

Accordingly, this study examined the associations between early postoperative SHR and four prespecified primary infection-related outcomes—suspected infection, culture-positive infection, Sepsis-3-defined sepsis, and septic shock—in critically ill patients after eligible cranial neurosurgical procedures. The secondary outcome was 90-day mortality; additional analyses assessed dose–response patterns, subgroup consistency, incremental discrimination and reclassification, temporal robustness, nested clinical subsets, and exploratory model-based indirect associations.

## 2. Methods

### 2.1. Study Design, Data Source, and Population

This retrospective study used data from the Medical Information Mart for Intensive Care IV (MIMIC-IV) database [23], which contains detailed clinical information on critically ill patients admitted to Beth Israel Deaconess Medical Center between 2008 and 2019. Access to the database was granted after completion of the National Institutes of Health “Protecting Human Research Participants” training and certification through the Collaborative Institutional Training Initiative program (Certification No. 68889220). The study was conducted in accordance with the Declaration of Helsinki and reported according to the STROBE statement.

Adults aged 18 years or older with an ICU admission and a qualifying cranial neurosurgical procedure were screened. No prespecified upper age limit was applied. To construct a patient-level cohort, only each patient’s first hospital admission and first ICU stay were eligible, and only the earliest eligible procedure was retained. Procedure records were linked using subject_id, hadm_id, and stay_id. Multiple qualifying procedure codes recorded on the same date were consolidated as one operative episode, and the end of the earliest eligible operation was defined as the index time. Subsequent ICU admissions and repeated eligible procedures did not generate additional observations. Patients were excluded for an ICU stay of 6 h or less, absence of an eligible procedure, pre-existing Sepsis-3-defined sepsis, unavailable data required to calculate SHR, missing essential timing data, or infection-related outcomes occurring on or before SHR assessment. After all linkage and exclusion steps, the final cohort contained 1408 unique subject_id, hadm_id, and stay_id values, with no duplicate patient records (Figure 1).

Eligible procedures were identified using prespecified ICD-9-CM and ICD-10-PCS codes (Supplementary Table S1). Standardized procedure descriptions were used to classify procedures into six mutually exclusive categories: cranial resection/biopsy, cerebrovascular procedure, intracranial evacuation/drainage, CSF diversion/drainage, skull-base/pituitary/cranial nerve procedure, and other cranial procedure. Because procedure descriptions do not reliably identify the underlying diagnosis or operative urgency, tumor- versus trauma-related indications and emergency versus elective surgery were not inferred. The resulting cohort therefore represents a heterogeneous postoperative neurocritical care population rather than a single diagnosis- or procedure-specific group. Procedure category was prespecified as a covariate in the multivariable analyses to account for measured differences among the broad surgical groups.

### 2.2. Assessment of Postoperative SHR

Postoperative glucose: The first serum glucose concentration obtained 6–24 h after the index procedure was used. A single early measurement was selected to preserve temporal ordering and reduce the influence of intraoperative fluctuations, subsequent treatment, nutritional support, and evolving complications. Because fasting status could not be verified, it was termed postoperative serum glucose rather than fasting glucose.

HbA1c selection: HbA1c measurements obtained within 90 days before surgery or during the index hospitalization before SHR assessment were eligible. When multiple values were available, the value closest to surgery was selected, prioritizing a preoperative measurement.

SHR calculation: Estimated average glucose (eAG) was calculated as eAG (mg/dL) = 28.7 × HbA1c (%) − 46.7. SHR was calculated as postoperative serum glucose (mg/dL)/eAG (mg/dL) and was dimensionless. Glucose values in mmol/L were converted to mg/dL by multiplying by 18.

Missing HbA1c: HbA1c was not imputed because it was a defining component of SHR; patients without an eligible measurement were excluded. Potential selection bias was assessed by comparing included and excluded patients and otherwise eligible patients with and without HbA1c measurements (Supplementary Tables S2 and S3).

Analytical classification: SHR was analyzed continuously and by quartiles. Glycemic status was classified as diabetes (documented diabetes or HbA1c ≥ 6.5%), prediabetes (HbA1c 5.7–6.4% without documented diabetes), or normal glucose regulation (HbA1c < 5.7% without documented diabetes).

### 2.3. Outcome Definitions

Suspected infection was defined by intravenous therapeutic antibiotic administration within 72 h after diagnostic culture collection or diagnostic culture collection within 24 h after antibiotic initiation. The index time was the earlier event. Routine perioperative prophylaxis was excluded. Qualifying bacterial or fungal cultures were obtained from blood, urine, respiratory tract, CSF, sterile body fluids, tissue, abscess, wound/deep tissue, or catheter tips; surveillance and other non-diagnostic tests were excluded. Culture-positive infection required growth of a potentially pathogenic organism from a qualifying specimen, and repeated cultures from the same episode were consolidated.

Suspected infection was an electronic health record (EHR)-derived indicator of clinical suspicion that prompted treatment and microbiological sampling, not a microbiologically confirmed diagnosis. Culture-positive infection remained susceptible to contamination and colonization. Because specimens came from multiple anatomical sites, both endpoints captured all-source postoperative infection-related events rather than neurosurgical surgical-site or intracranial infections. “Postoperative” denotes timing after the index procedure and does not establish surgery as the source.

Sepsis-3-defined sepsis was defined as suspected infection accompanied by an acute increase in SOFA score of at least 2 points from the admission baseline. Septic shock was defined using a strict Sepsis-3-consistent EHR proxy: Sepsis-3-defined sepsis plus vasopressor therapy, MAP < 65 mmHg, and lactate > 2 mmol/L within the same 24-h assessment window after sepsis onset. The MAP and lactate values closest to vasopressor initiation were selected. Because adequacy of fluid resuscitation could not be adjudicated, this endpoint was considered an EHR-based proxy rather than a fully clinician-adjudicated diagnosis. The secondary outcome was all-cause mortality within 90 days after the index procedure.

### 2.4. Covariates

Covariates were selected a priori according to clinical relevance and data availability, emphasizing baseline confounders and excluding post-exposure variables, potential mediators, and outcome components. Model 1 was adjusted for age, sex, race, and heart rate. Model 2 was additionally adjusted for chronic kidney disease, hypertension, atrial fibrillation, diabetes mellitus, congestive heart failure, hyperlipidemia, anemia, and procedure category. Model 3 was further adjusted for red blood cell count, platelet count, hemoglobin, hematocrit, bicarbonate, blood urea nitrogen, calcium, chloride, creatinine, sodium, potassium, international normalized ratio, prothrombin time, partial thromboplastin time, GCS, and the Charlson Comorbidity Index. Diabetes was included because it may confound associations between SHR and infection-related outcomes. The GCS variable was treated as an available ICU-recorded measure of neurological status rather than as a uniformly defined preoperative assessment.

For all other retained covariates, pre-imputation missingness was below 20%; variables with missingness of 20% or more were excluded from multivariable modeling. Missing values in retained covariates were imputed with missForest, leaving no missing modeled values [24]. No propensity score matching, inverse probability weighting, or other covariate-balancing procedure was applied. Table 1 presents unmatched and unweighted descriptive data from the completed analytic dataset after missForest imputation.

### 2.5. Statistical Analysis

Continuous variables are reported as the mean ± standard deviation or median (interquartile range), and categorical variables are reported as the number (or percentage). Groups were compared using analysis of variance or Kruskal–Wallis tests and chi-square or Fisher exact tests, as appropriate.

Multivariable logistic regression estimated odds ratios (ORs) and 95% confidence intervals (CIs) for SHR were modeled continuously and by quartiles. Restricted cubic splines with four knots at the 5th, 35th, 65th, and 95th percentiles were assessed nonlinearity; the median SHR was the reference. Overall and nonlinear associations were evaluated using Wald tests. Prespecified subgroup analyses used the primary adjustment set, excluding the stratification variable. Interaction *p*-values were obtained from multiplicative terms between continuous SHR and subgroup variables and were considered exploratory.

Boruta analyses were performed separately for each infection-related outcome using the Boruta package and normalized random-forest importance (getImpRfZ). Each forest contained 500 trees; maxRuns was 500; the *p*-value was 0.01 with Bonferroni correction; a fixed seed was used; and unresolved variables remained tentative. Correlated predictors were retained because Boruta seeks all relevant features; therefore, importance among correlated variables was interpreted as shared or redundant relevance rather than independent contribution. Boruta was used only for exploratory ranking and did not determine covariate selection.

Incremental performance was assessed by adding SHR to the clinical model and comparing AUCs using the DeLong test; category-free NRI and IDI were estimated with bootstrap CIs. Exploratory nested analyses evaluated associations across progressively severe clinical states. A 24-h lag sensitivity analysis excluded patients who developed the corresponding outcome within 24 h after SHR assessment and counted only later incident events.

Exploratory mediation analyses evaluated Sepsis-3-defined sepsis and septic shock as potential indirect pathways between standardized SHR and 90-day mortality within a regression-based counterfactual framework. Only mediator events occurring after SHR assessment and before death were included. ACME, ADE, total effect, and estimated indirect proportion were reported on the absolute probability scale with 95% CIs from 5000 nonparametric bias-corrected and accelerated bootstrap resamples. Because the assumptions required for causal mediation cannot be verified in this observational cohort, these results were interpreted as model-based indirect associations rather than causal effects.

All analyses were performed in R version 4.3.1 (R Foundation for Statistical Computing, Vienna, Austria). Tests were two-sided, with *p* < 0.05 considered statistically significant. Exploratory analyses were interpreted primarily using effect estimates and 95% CIs.

## 3. Results

### 3.1. Study Population and Baseline Characteristics

Among 94,458 patients screened in the intensive care unit module, 1408 met the eligibility criteria and were included in the final analysis (Figure 1). The mean age was 62.56 ± 15.71 years (range, 18.0–98.0 years), 687 patients (48.79%) were female, and 377 (26.78%) had pre-existing diabetes mellitus. Among patients with pre-existing diabetes mellitus, the median HbA1c was 7.09% (IQR, 6.30–8.30%); 174 (46.15%) had an HbA1c < 7.0%, 85 (22.55%) had an HbA1c of 7.0% to <8.0%, and 118 (31.30%) had an HbA1c ≥ 8.0%. Across increasing SHR quartiles, patients had higher heart rate, a greater prevalence of diabetes, congestive heart failure, and anemia, higher white blood cell count, lactate, potassium, and postoperative glucose concentrations, and lower lymphocyte percentages. Higher SHR was also accompanied by greater illness severity, as reflected by higher SOFA, SAPS II, OASIS, and Charlson Comorbidity Index values, together with more frequent mechanical ventilation and longer hospital and ICU stays. Procedure categories differed significantly across SHR quartiles (Table 1).

Overall, 540 patients (38.35%) met the suspected-infection definition, 225 (15.98%) had culture-positive infection, 369 (26.21%) met the Sepsis-3 definition, and 148 (10.51%) met the strict EHR-based septic shock proxy. The incidence of each infection-related outcome increased across SHR quartiles: suspected infection increased from 31.07% in Q1 to 54.26% in Q4, culture-positive infection increased from 10.73% to 25.28%, Sepsis-3-defined sepsis increased from 16.95% to 42.61%, and septic shock increased from 4.52% to 19.89% (all *p* < 0.001). Mortality also increased across quartiles; 90-day mortality was 3.11% in Q1 and 12.50% in Q4 (*p* < 0.001) (Table 1). Patients who met the Sepsis-3 criteria had greater physiological derangement, longer ICU and hospital stays, and higher 90-day mortality than those who did not meet these criteria (14.63% vs. 3.08%; Supplementary Table S4).

Culture positivity and Sepsis-3-defined sepsis overlapped but were not identical. Among the 369 patients meeting the Sepsis-3 criteria, 166 (45.0%) had a qualifying positive culture, whereas 203 (55.0%) did not. Conversely, 59 of the 225 culture-positive patients (26.2%) did not meet the Sepsis-3 criteria. Thus, the culture-positive infection and Sepsis-3-defined sepsis counts represent non-mutually exclusive features within the 540-patient suspected-infection cohort.

Comparisons undertaken to assess potential selection bias showed that patients included in the final cohort were older and had a higher prevalence of several cardiometabolic comorbidities than excluded patients, whereas 90-day mortality was similar (6.11% vs. 6.07%, *p* = 1.000; Supplementary Table S2). Likewise, otherwise eligible patients with an available HbA1c measurement were older and more frequently had diabetes, hypertension, and hyperlipidemia than those without HbA1c, but 90-day mortality did not differ significantly (6.11% vs. 7.29%, *p* = 0.280; Supplementary Table S3). The mean interval from the end of the index procedure to SHR assessment was 12.68 h. Among patients with suspected infection, the median interval from SHR assessment to the suspected-infection index time was 17.0 h. Among patients meeting the Sepsis-3-defined sepsis criteria, the corresponding interval was 30.4 h.

### 3.2. Associations Between SHR and Infection-Related Outcomes

Higher postoperative SHR was associated with all four infection-related outcomes in multivariable logistic regression analyses. In Model 3, each 0.1-unit increase in SHR was associated with higher odds of suspected infection (OR, 1.08; 95% CI, 1.04–1.12; *p* < 0.001), culture-positive infection (OR, 1.05; 95% CI, 1.01–1.09; *p* = 0.010), Sepsis-3-defined sepsis (OR, 1.07; 95% CI, 1.03–1.11; *p* < 0.001), and septic shock (OR, 1.10; 95% CI, 1.05–1.15; *p* < 0.001) (Table 2).

The quartile analyses showed a graded increase in risk. Compared with Q1, Q4 was associated with higher odds of suspected infection (OR, 1.98; 95% CI, 1.41–2.78), culture-positive infection (OR, 1.87; 95% CI, 1.20–2.91), Sepsis-3-defined sepsis (OR, 2.89; 95% CI, 1.98–4.23), and septic shock (OR, 2.90; 95% CI, 1.56–5.40). Higher odds of Sepsis-3-defined sepsis were also observed in Q3 (OR, 1.54; 95% CI, 1.03–2.29), whereas the odds of septic shock were elevated from Q2 onward (Q2: OR, 2.02; 95% CI, 1.03–3.96; Q3: OR, 2.19; 95% CI, 1.14–4.21).

### 3.3. Feature Importance and Dose–Response Relationship

In the exploratory Boruta analysis, SHR was classified as a confirmed relevant feature for each infection-related outcome and ranked among the more influential features for Sepsis-3-defined sepsis and septic shock. Because Boruta is an all-relevant feature-selection method and correlated predictors may share importance, these findings were interpreted as model-based feature relevance rather than as independent effect estimates or evidence of causal importance (Figure 2).

Restricted cubic spline analyses demonstrated significant overall associations between SHR and suspected infection, culture-positive infection, Sepsis-3-defined sepsis, and septic shock (all *p*-values for overall association were <0.001). The association with suspected infection was approximately linear (*p*-value for nonlinearity = 0.132). A significant nonlinear relationship was observed for culture-positive infection (*p*-value for nonlinearity = 0.005), characterized by a steeper increase at lower-to-moderate SHR values and a flatter slope at higher values. The associations with Sepsis-3-defined sepsis and septic shock were predominantly monotonic, with *p*-values for nonlinearity of 0.053 and 0.068, respectively (Figure 3).

### 3.4. Subgroup and Stratified Analyses

The direction of association between SHR and infection-related outcomes was generally positive across clinically relevant subgroups, although several interaction tests indicated heterogeneity (Figure 4). For suspected infection, significant interactions were observed for age (*p* for interaction < 0.001), hyperlipidemia (*p* = 0.001), and diabetes mellitus (*p* = 0.005). For culture-positive infection, an interaction was observed for acute kidney injury (*p* = 0.010). The association with Sepsis-3-defined sepsis varied by age (*p* = 0.002), diabetes mellitus (*p* = 0.010), and Glasgow Coma Scale category (*p* = 0.040). For septic shock, significant interactions were observed for chronic kidney disease (*p* = 0.041) and Glasgow Coma Scale category (*p* = 0.049). Because multiple subgroup comparisons were performed, these findings were considered exploratory.

In analyses stratified by glycemic status, higher SHR remained associated with several infection-related outcomes across normal glucose regulation, prediabetes, and diabetes (Supplementary Figures S1 and S2, and Table S7). In the normal-glucose-regulation subgroup, Q4 was associated with all four outcomes. In the prediabetes subgroup, Q4 was associated with suspected infection, Sepsis-3-defined sepsis, and septic shock, whereas the association with culture-positive infection was not statistically significant. In the diabetes subgroup, Q4 was associated with all four outcomes, including culture-positive infection (OR, 2.84; 95% CI, 1.41–5.74) and septic shock (OR, 2.96; 95% CI, 1.49–5.88).

### 3.5. Incremental Discrimination and Reclassification Analyses

Adding SHR to the clinical model produced small increases in discrimination that did not reach statistical significance for suspected infection (AUC, 0.774 to 0.776; *p* = 0.337), culture-positive infection (0.777 to 0.778; *p* = 0.401), Sepsis-3-defined sepsis (0.810 to 0.814; *p* = 0.067), or septic shock (0.796 to 0.804; *p* = 0.077). For suspected infection, the IDI was 0.004 (95% CI, 0.001–0.007; *p* = 0.014), whereas the NRI was not statistically significant. No significant improvement in NRI or IDI was observed for culture-positive infection. In contrast, addition of SHR improved both NRI and IDI for Sepsis-3-defined sepsis (NRI, 0.172; 95% CI, 0.056–0.290; *p* = 0.004; IDI, 0.007; 95% CI, 0.002–0.012; *p* = 0.002) and septic shock (NRI, 0.206; 95% CI, 0.043–0.372; *p* = 0.015; IDI, 0.010; 95% CI, 0.002–0.019; *p* = 0.018) (Table 3). These incremental discrimination and reclassification analyses were considered exploratory.

### 3.6. Associations Within Nested Clinical Subsets

In analyses of nested clinical subsets, higher SHR was associated with suspected infection in the overall cohort (Model 3 OR, 1.32; 95% CI, 1.16–1.49; *p* < 0.001). Among patients with suspected infection, SHR was not associated with culture-positive infection (OR, 1.00; 95% CI, 0.84–1.17; *p* = 0.958), but remained associated with Sepsis-3-defined sepsis (OR, 1.31; 95% CI, 1.07–1.62; *p* = 0.012). Among patients with Sepsis-3-defined sepsis, higher SHR was associated with septic shock (OR, 1.36; 95% CI, 1.11–1.68; *p* = 0.004). Higher SHR was also associated with 90-day mortality in the overall cohort (OR, 1.59; 95% CI, 1.29–1.96; *p* < 0.001), among patients with Sepsis-3-defined sepsis (OR, 1.41; 95% CI, 1.06–1.86; *p* = 0.016), and among those with septic shock (OR, 1.63; 95% CI, 1.04–2.66; *p* = 0.039) (Table 4). These analyses describe associations within progressively restricted clinical subsets and do not establish a causal sequence of disease progression.

### 3.7. Sensitivity and Exploratory Mediation Analyses

The associations remained directionally consistent in the 24-h lag analysis. After excluding patients who developed the corresponding outcome within 24 h after SHR assessment, each 0.1-unit increase in SHR remained associated with suspected infection (OR, 1.08; 95% CI, 1.04–1.12), culture-positive infection (OR, 1.05; 95% CI, 1.01–1.10), Sepsis-3-defined sepsis (OR, 1.10; 95% CI, 1.06–1.15), and septic shock (OR, 1.13; 95% CI, 1.07–1.19) in Model 3. Similarly, Q4 versus Q1 remained associated with suspected infection (OR, 1.90; 95% CI, 1.33–2.71), culture-positive infection (OR, 2.20; 95% CI, 1.37–3.53), Sepsis-3-defined sepsis (OR, 1.92; 95% CI, 1.23–3.00), and septic shock (OR, 3.91; 95% CI, 1.86–8.25) (Supplementary Table S5).

In exploratory mediation models, the estimated average indirect association through subsequent Sepsis-3-defined sepsis was 0.0070 (95% CI, 0.0029–0.0138), corresponding to an estimated indirect proportion of 19.7% (95% CI, 8.6–37.8%). The estimated average indirect association through septic shock was 0.0054 (95% CI, 0.0010–0.0094), with an estimated indirect proportion of 14.8% (95% CI, 2.9–28.0%) (Supplementary Table S6). These estimates were interpreted as model-based indirect associations rather than evidence of causal mediation.

## 4. Discussion

In this cohort of 1408 critically ill patients after cranial neurosurgical procedures, higher early postoperative SHR was associated with all four infection-related outcomes and 90-day mortality. Associations were strongest for Sepsis-3-defined sepsis and septic shock, weaker for culture-positive infection, and directionally consistent in the 24-h lag analysis. Adding SHR minimally changed AUC, although reclassification improved for Sepsis-3-defined sepsis and septic shock. SHR may therefore provide complementary risk information, but these associations do not establish that it predicts neurosurgical infection.

The present findings are consistent with the growing literature linking relative hyperglycemia to adverse outcomes in acute and critical illness [25,26]. SHR contextualizes acute glucose against the patient’s chronic glycemic background and may therefore distinguish an acute metabolic stress response from chronically elevated glucose more effectively than an isolated glucose value [8]. This distinction is particularly relevant in mixed ICU populations that include patients with diabetes, prediabetes, and previously unrecognized dysglycemia. Prior neurosurgical studies have linked perioperative hyperglycemia or elevated HbA1c to postoperative infection after craniotomy, while studies in spine and cardiac surgery have similarly associated postoperative hyperglycemia with surgical-site infection and other adverse outcomes [27,28,29,30,31,32,33,34,35]. Our results extend this literature by evaluating relative rather than absolute hyperglycemia and by examining several clinically distinct infection-related outcomes within the same neurocritical care cohort.

Differences in effect size across outcomes warrant caution. Culture positivity depends on specimen type, sampling intensity, timing relative to antimicrobial exposure, contamination, colonization, and local practice [36,37]. Sepsis-3-defined sepsis incorporates acute organ dysfunction, while the septic shock proxy also captures circulatory and metabolic abnormalities [22]. Stronger associations with these outcomes may reflect overall host response rather than microbiological confirmation. The short median intervals from SHR assessment to suspected infection (17.0 h) and Sepsis-3-defined sepsis (30.4 h) raise the possibility that occult infection or an evolving inflammatory response was already present when SHR was measured [10,11]. Because recorded onset represented the first fulfillment of the EHR criteria rather than biological onset, elevated SHR may partly reflect an ongoing inflammatory process. Although the 24-h lag analysis reduced temporal overlap, it could not exclude residual reverse causation or establish the temporal direction of the observed associations.

Suspected infection indicates clinical suspicion rather than confirmed infection. Culture positivity provides microbiological evidence but cannot distinguish infection from colonization or contamination. Because specimens were collected from multiple anatomical sites, neither endpoint specifically represents neurosurgical surgical-site or intracranial infection. The findings therefore concern all-source infection-related events in the postoperative neurocritical care setting.

Among patients meeting the Sepsis-3 criteria, 203 of 369 (55.0%) did not have a qualifying positive culture. Potential explanations include antimicrobial exposure before specimen collection; limitations in the timing, anatomical site, and sensitivity of microbiological sampling; low organism burden; or difficult-to-culture pathogens. In addition, the EHR-based definition may classify patients with suspected infection and acute organ dysfunction caused partly or wholly by noninfectious conditions. Because the retrospective data did not permit case-level adjudication of these possibilities, these cases should be interpreted as Sepsis-3-defined events without microbiological confirmation rather than as definitively proven culture-negative infections.

Associations were observed across normal glucose regulation, prediabetes, and diabetes, but subgroup findings remain exploratory because of multiple comparisons and limited event counts. The prognostic meaning of acute hyperglycemia may vary with chronic glycemic status [34,38], but these findings do not define subgroup-specific treatment thresholds or justify different glycemic targets. SHR is not a diagnostic test and should not independently trigger antimicrobial or insulin therapy. The modest AUC changes suggest that it cannot replace clinical assessment, although it may prompt closer review of glucose trends, infection-related findings, organ function, and hemodynamic status. Observational neurosurgical studies suggest benefits from structured perioperative glucose management [28], whereas critical-care trials show that intensive glucose lowering can cause hypoglycemia and worsen outcomes [39,40,41,42,43]. Prospective studies must determine whether SHR-guided surveillance or treatment improves outcomes.

The exploratory mediation analyses should also be interpreted with restraint. The observed indirect associations through subsequent Sepsis-3-defined sepsis and septic shock are compatible with, but do not prove, a pathway linking postoperative metabolic stress to mortality. Causal mediation requires no unmeasured confounding of the exposure–mediator, mediator–outcome, and exposure–outcome relationships, correct temporal ordering, and correct model specification. These assumptions are unlikely to be fully satisfied in a retrospective ICU cohort in which metabolic, infectious, and organ-dysfunction processes evolve simultaneously. The mediation results should therefore be regarded as descriptive, model-dependent, and hypothesis-generating.

Several limitations should be acknowledged. First, SHR required clinically available HbA1c, and testing was unlikely to be random. Patients with HbA1c were older and more often had diabetes, hypertension, and hyperlipidemia than eligible patients without HbA1c. Similar 90-day mortality did not eliminate selection bias; generalizability may therefore be limited to patients with available HbA1c. Second, EHR outcome times reflected first criterion fulfillment rather than biological onset, so the 24-h lag analysis could not exclude residual reverse causation. Third, suspected infection was an EHR-derived surrogate, while culture-positive infection remained sensitive to sampling, prior antibiotics, contamination, colonization, and culture-negative disease [36,37]. The septic shock proxy could not confirm adequate fluid resuscitation, and vasopressors in neurocritical care may support cerebral perfusion rather than treat distributive shock [44].

Fourth, unavailable perioperative factors—including corticosteroids, insulin treatment, nutritional glucose load, surgical urgency and indication, operative duration, transfusion, CSF leakage, and drain duration—may have caused residual confounding. Accordingly, adjusted associations should not be interpreted as independent causal effects. HbA1c may also be distorted by anemia, blood loss, transfusion, renal dysfunction, or altered erythrocyte turnover [45]. Finally, practice changed during 2008–2019, and exploratory analyses were susceptible to multiplicity and model dependence. Because the cohort included six broad procedure categories and MIMIC-IV uses patient-specific shifted dates, cohort size should not be interpreted as the annual volume of conventional craniotomy [23]. Local referral, coding, and ICU admission practices may limit generalizability. Residual confounding across procedure categories also remains possible, so estimates should be interpreted at the cohort level.

## 5. Conclusions

Higher early postoperative SHR was associated with EHR-derived, all-source infection-related and sepsis-related events and 90-day mortality after cranial neurosurgical procedures. These associations do not establish prediction of neurosurgical infection; prospective external validation is required before SHR informs surveillance or glycemic intervention.

## Figures and Tables

**Figure 1:**
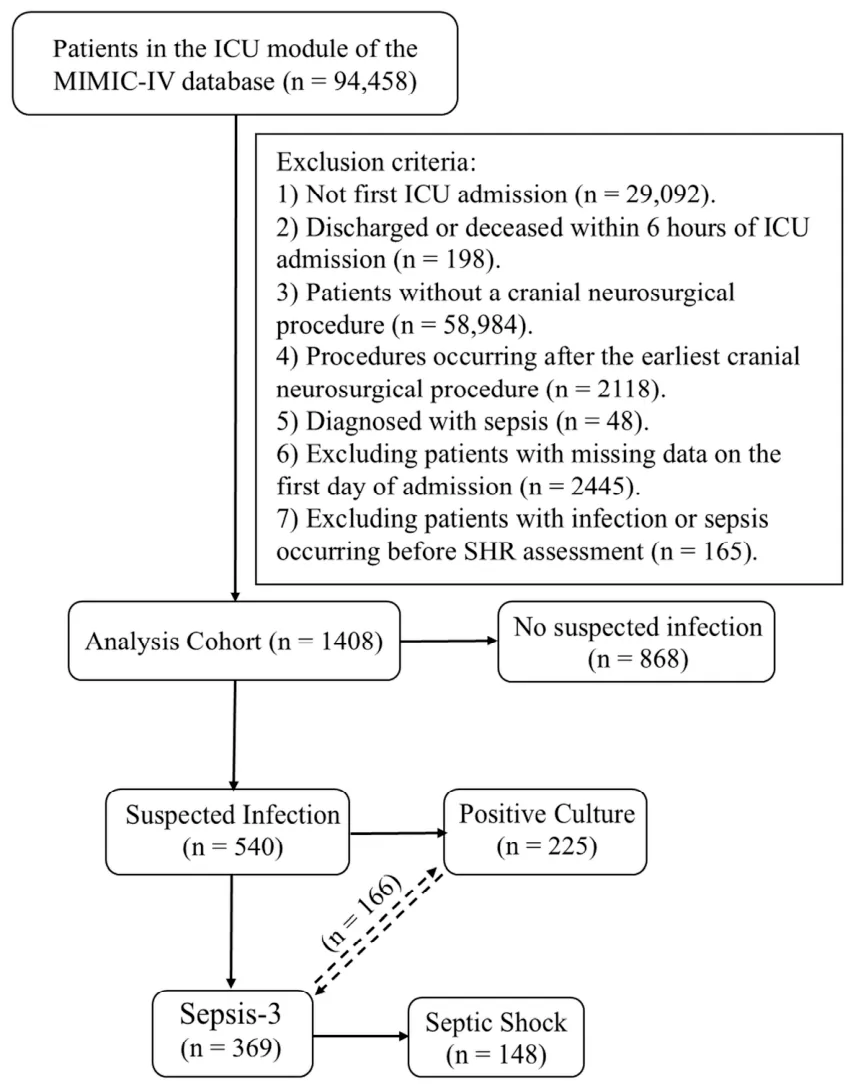
Flowchart of patient selection in the MIMIC-IV database. Solid arrows indicate cohort flow; the dashed bidirectional arrow denotes the overlap between culture-positive infection and Sepsis-3-defined sepsis (*n* = 166).

**Figure 2:**
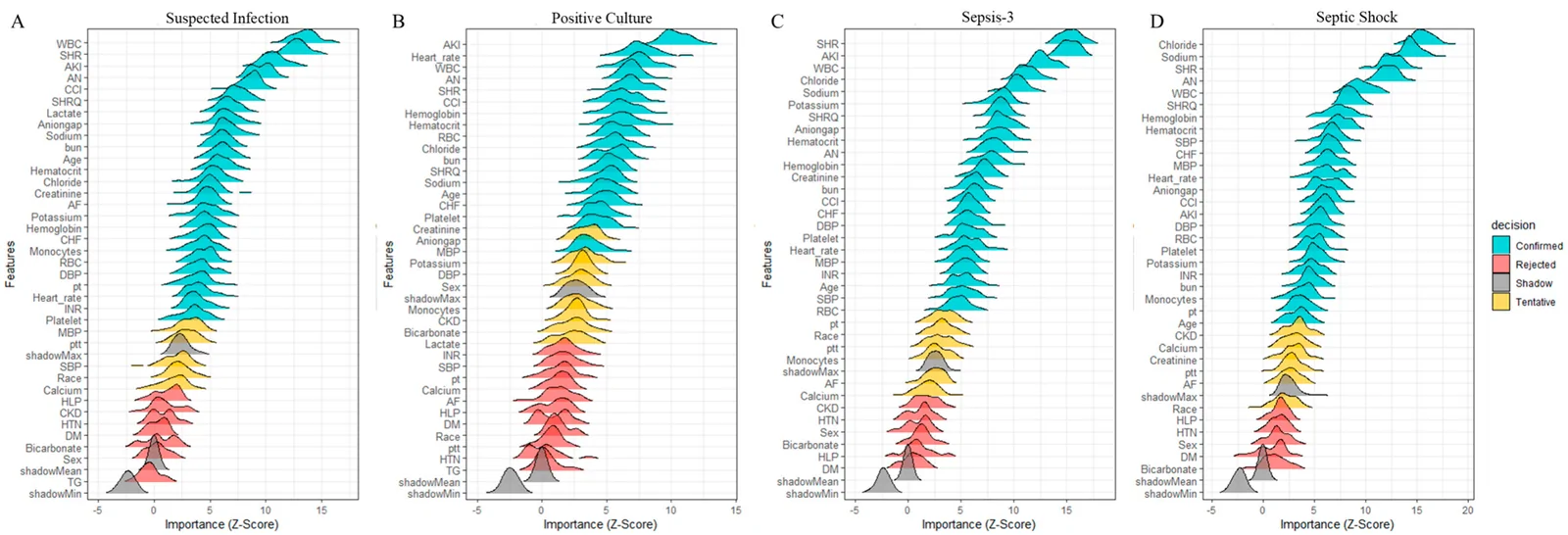
Boruta-based feature importance ridge plot for infection-related outcomes in patients after cranial neurosurgical procedures: (**A**) suspected infection; (**B**) culture-positive infection; (**C)** Sepsis-3-defined sepsis; and (**D**) septic shock.

**Figure 3:**
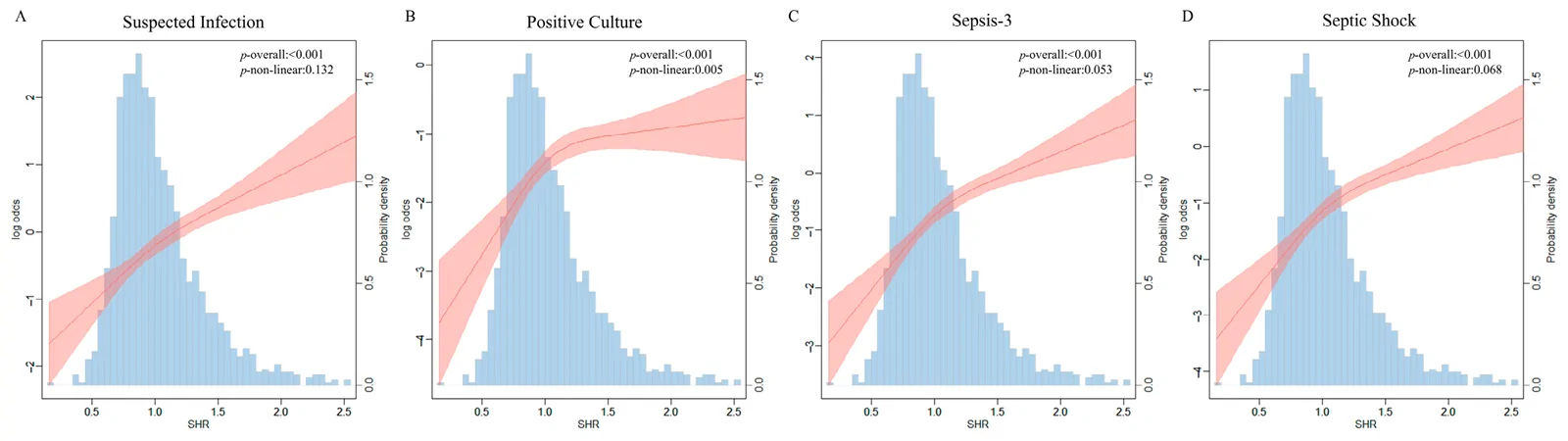
Dose–response relationships between SHR and infection-related outcomes in patients after cranial neurosurgical procedures ((**A**) Suspected Infection; (**B**) Positive Culture; (**C**) Sepsis-3; (**D**) Septic Shock). Restricted cubic spline models were fitted using four knots placed at the 5th, 35th, 65th, and 95th percentiles of the SHR distribution. Models were adjusted for age, sex, race, weight, HR, DM, CCI, GCS, and procedure category. SHR, stress hyperglycemia ratio; HR, heart rate; DM, diabetes mellitus; CCI, Charlson Comorbidity Index; GCS, Glasgow Coma Scale.

**Figure 4:**
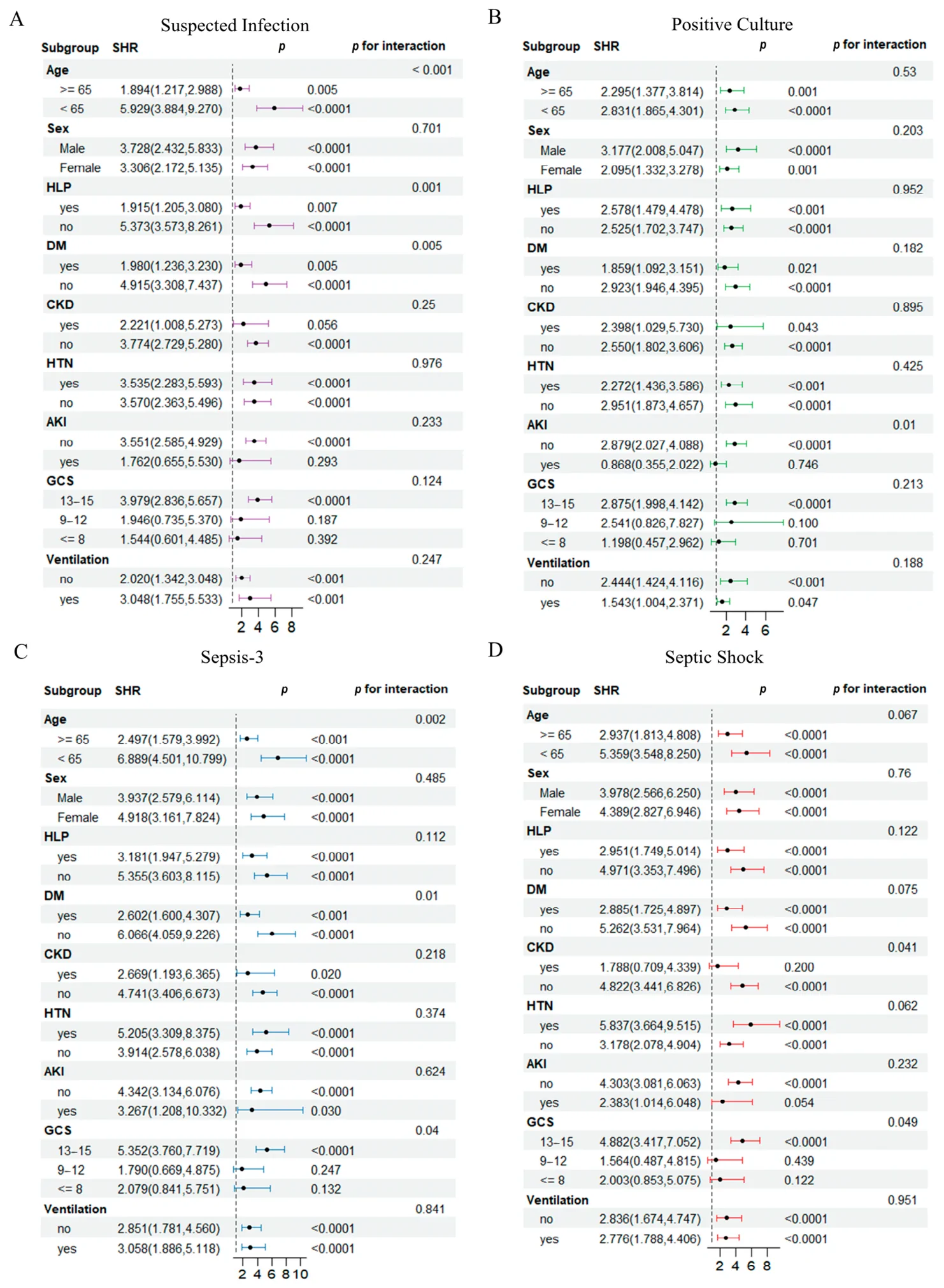
Subgroup analyses of the associations between SHR and infection-related outcomes in patients after cranial neurosurgical procedures: (**A**) suspected infection; (**B**) culture-positive infection; (**C**) Sepsis-3-defined sepsis; and (**D**) septic shock. Each panel presents adjusted odds ratios and 95% confidence intervals across clinically relevant subgroups. Models were adjusted for age, sex, race, weight, HR, DM, CCI, GCS, and procedure category, excluding the corresponding stratification variable from each subgroup-specific model. SHR, stress hyperglycemia ratio; HR, heart rate; DM, diabetes mellitus; CCI, Charlson Comorbidity Index; GCS, Glasgow Coma Scale.

**Table 1:** Baseline characteristics and clinical outcomes according to SHR quartile.

Variables	Total (*n* = 1408)	Q1 (*n* = 354)	Q2 (*n* = 350)	Q3 (*n* = 352)	Q4 (*n* = 352)	*p*
Demographics						
Age, years	62.56 ± 15.71	61.73 ± 15.41	62.47 ± 14.95	63.66 ± 16.70	62.38 ± 15.72	0.430
Sex, female, *n* (%)	687 (48.79)	195 (55.08)	162 (46.29)	163 (46.31)	167 (47.44)	0.050
Weight, kg	82.17 ± 21.40	79.48 ± 19.87	82.98 ± 19.06	82.22 ± 22.01	84.03 ± 24.11	0.030
Race						0.100
Other, *n* (%)	633 (44.96)	161 (45.48)	155 (44.29)	142 (40.34)	175 (49.72)	
White, *n* (%)	775 (55.04)	193 (54.52)	195 (55.71)	210 (59.66)	177 (50.28)	
Vital signs						
HR mean, beats/min	79.97 ± 13.69	78.95 ± 13.99	78.54 ± 12.31	80.32 ± 13.66	82.07 ± 14.48	<0.010
SBP mean, mmHg	127.54 ± 13.11	126.45 ± 12.33	126.94 ± 12.47	128.13 ± 13.77	128.63 ± 13.73	0.100
DBP mean, mmHg	66.57 ± 10.20	67.01 ± 10.04	66.45 ± 9.17	66.85 ± 10.89	65.97 ± 10.62	0.530
MBP mean, mmHg	85.21 ± 9.41	85.74 ± 9.27	84.96 ± 8.56	85.36 ± 9.96	84.78 ± 9.80	0.530
Comorbidity						
CKD, *n* (%)	118 (8.38)	33 (9.32)	24 (6.86)	29 (8.24)	32 (9.09)	0.640
HTN, *n* (%)	736 (52.27)	179 (50.56)	181 (51.71)	189 (53.69)	187 (53.13)	0.840
AF, *n* (%)	147 (10.44)	24 (6.78)	36 (10.29)	47 (13.35)	40 (11.36)	0.040
DM, *n* (%)	377 (26.78)	81 (22.88)	72 (20.57)	98 (27.84)	126 (35.80)	<0.001
CHF, *n* (%)	110 (7.81)	19 (5.37)	20 (5.71)	33 (9.38)	38 (10.80)	0.010
HLP, *n* (%)	601 (42.68)	143 (40.40)	143 (40.86)	163 (46.31)	152 (43.18)	0.370
AN, *n* (%)	221 (15.70)	41 (11.58)	39 (11.14)	60 (17.05)	81 (23.01)	<0.001
AKI, *n* (%)	101 (7.17)	19 (5.37)	18 (5.14)	32 (9.09)	32 (9.09)	0.050
Laboratory indicators						
RBC (10^12^/L)	4.45 ± 0.61	4.50 ± 0.57	4.54 ± 0.52	4.39 ± 0.63	4.37 ± 0.69	<0.001
WBC (10^9^/L)	9.37 ± 4.32	8.06 ± 3.60	8.41 ± 3.38	9.47 ± 3.94	11.57 ± 5.24	<0.001
Platelet (10^9^/L)	241.13 ± 75.26	249.62 ± 71.87	242.73 ± 71.57	239.83 ± 78.71	232.32 ± 77.88	0.020
Hemoglobin (g/dL)	13.36 ± 1.77	13.40 ± 1.62	13.68 ± 1.62	13.24 ± 1.84	13.10 ± 1.93	<0.010
Hematocrit (%)	37.45 ± 5.20	36.90 ± 5.06	37.60 ± 5.05	37.29 ± 5.34	38.01 ± 5.32	0.030
HbA1c (%)	6.24 ± 1.33	6.52 ± 1.54	6.07 ± 1.00	6.13 ± 1.32	6.22 ± 1.38	<0.001
Monocytes (%)	6.33 ± 2.70	6.67 ± 2.47	6.44 ± 2.53	6.48 ± 2.82	5.75 ± 2.88	<0.001
Lymphocytes (%)	20.67 ± 11.57	25.12 ± 10.83	23.18 ± 11.26	19.56 ± 11.47	14.81 ± 9.93	<0.001
Anion gap (mmol/L)	15.06 ± 3.51	14.95 ± 3.24	14.67 ± 3.11	14.85 ± 3.79	15.78 ± 3.76	<0.001
Bicarbonate (mmol/L)	23.60 ± 2.96	23.55 ± 2.90	23.75 ± 2.75	23.45 ± 2.98	23.65 ± 3.18	0.570
BUN (mg/dL)	17.03 ± 9.42	16.66 ± 9.43	15.71 ± 7.59	17.50 ± 10.72	18.27 ± 9.52	<0.010
Calcium (mg/dL)	8.74 ± 0.61	8.76 ± 0.59	8.72 ± 0.57	8.71 ± 0.64	8.77 ± 0.62	0.460
Chloride (mmol/L)	106.33 ± 5.21	106.42 ± 5.19	106.30 ± 4.58	106.28 ± 4.89	106.30 ± 6.09	0.980
Creatinine (mg/dL)	1.00 ± 0.89	0.99 ± 0.87	0.94 ± 0.68	1.04 ± 1.10	1.05 ± 0.84	0.380
Lactate (mmol/L)	1.96 ± 1.44	1.77 ± 1.01	1.73 ± 1.36	1.97 ± 1.36	2.36 ± 1.81	<0.001
Sodium (mmol/L)	140.99 ± 4.51	141.07 ± 4.48	140.85 ± 3.78	140.77 ± 4.26	141.27 ± 5.38	0.450
Potassium (mmol/L)	4.20 ± 0.67	4.13 ± 0.61	4.11 ± 0.58	4.23 ± 0.68	4.33 ± 0.77	<0.001
TG (mg/dL)	155.33 ± 112.00	148.30 ± 85.86	144.00 ± 71.52	158.15 ± 116.05	170.83 ± 154.56	<0.010
Glucose (mg/dL)	134.53 ± 59.67	94.62 ± 25.24	110.46 ± 24.57	135.86 ± 41.12	197.29 ± 72.15	<0.001
INR	1.27 ± 0.82	1.26 ± 0.51	1.22 ± 0.57	1.38 ± 1.43	1.23 ± 0.26	0.040
PT (seconds)	13.97 ± 8.26	13.74 ± 5.32	13.37 ± 4.98	15.27 ± 14.50	13.49 ± 2.78	<0.010
PTT (seconds)	33.32 ± 19.15	32.85 ± 18.21	33.33 ± 19.26	33.32 ± 19.22	33.77 ± 19.95	0.940
Disease severity score						
SOFA	2.62 ± 2.19	2.40 ± 2.10	2.31 ± 1.95	2.67 ± 2.20	3.10 ± 2.43	<0.001
SAPS II	29.34 ± 11.07	28.84 ± 11.03	27.48 ± 10.14	29.39 ± 10.80	31.62 ± 11.89	<0.001
OASIS	27.35 ± 8.18	25.44 ± 7.69	25.66 ± 7.21	27.91 ± 8.30	30.41 ± 8.48	<0.001
GCS	13.22 ± 2.70	13.38 ± 2.44	13.44 ± 2.38	13.14 ± 2.65	12.93 ± 3.22	0.050
CCI	4.13 ± 2.52	3.90 ± 2.55	3.90 ± 2.44	4.30 ± 2.61	4.43 ± 2.44	<0.010
Treatment						
Ventilation, *n* (%)	441 (31.32)	78 (22.03)	88 (25.14)	111 (31.53)	164 (46.59)	<0.001
Vasoactive drug, *n* (%)	214 (15.20)	46 (12.99)	47 (13.43)	51 (14.49)	70 (19.89)	0.040
Procedure category						<0.001
Cerebrovascular procedure	304 (21.59)	82 (23.16)	97 (27.71)	67 (19.03)	58 (16.48)	
Cranial resection/biopsy	383 (27.20)	111 (31.36)	110 (31.43)	95 (26.99)	67 (19.03)	
CSF diversion/drainage	186 (13.21)	29 (8.19)	36 (10.29)	37 (10.51)	84 (23.86)	
Intracranial evacuation/drainage	146 (10.37)	46 (12.99)	30 (8.57)	36 (10.23)	34 (9.66)	
Other cranial procedure	244 (17.33)	51 (14.41)	48 (13.71)	73 (20.74)	72 (20.45)	
Skull-base/pituitary/cranial nerve procedure	145 (10.30)	35 (9.89)	29 (8.29)	44 (12.50)	37 (10.51)	
Infection-related outcomes						
Suspected infection	540 (38.35)	110 (31.07)	107 (30.57)	132 (37.50)	191 (54.26)	<0.001
Culture-positive infection	225 (15.98)	38 (10.73)	43 (12.29)	55 (15.63)	89 (25.28)	<0.001
Sepsis-3	369 (26.21)	60 (16.95)	71 (20.29)	88 (25.00)	150 (42.61)	<0.001
Septic shock	148 (10.51)	16 (4.52)	26 (7.43)	36 (10.23)	70 (19.89)	<0.001
Length of stay (LOS)						
LOS in hospital	11.24 ± 11.21	9.19 ± 9.31	9.68 ± 9.54	11.58 ± 11.92	14.49 ± 12.89	<0.001
LOS in ICU	5.71 ± 6.93	4.11 ± 4.78	4.83 ± 6.56	5.99 ± 7.41	7.89 ± 7.97	<0.001
Outcomes						
ICU death, *n* (%)	80 (5.68)	11 (3.11)	10 (2.86)	17 (4.83)	42 (11.93)	<0.001
In-hospital death, *n* (%)	96 (6.82)	14 (3.95)	14 (4.00)	22 (6.25)	46 (13.07)	<0.001
30-day mortality, *n* (%)	84 (5.97)	11 (3.11)	10 (2.86)	19 (5.40)	44 (12.50)	<0.001
90-day mortality, *n* (%)	86 (6.11)	11 (3.11)	11 (3.14)	20 (5.68)	44 (12.50)	<0.001

SHR quartiles were Q1 (0.16–0.78), Q2 (0.78–0.94), Q3 (0.94–1.19), and Q4 (1.19–2.98). SHR, stress hyperglycemia ratio; HR, heart rate; SBP, systolic blood pressure; DBP, diastolic blood pressure; MBP, mean blood pressure; CKD, chronic kidney disease; HTN, hypertension; AF, atrial fibrillation; DM, diabetes mellitus; CHF, congestive heart failure; HLP, hyperlipidemia; AN, anemia; AKI, acute kidney injury; RBC, red blood cell count; WBC, white blood cell count; HbA1c, glycated hemoglobin; BUN, blood urea nitrogen; TG, triglycerides; INR, international normalized ratio; PT, prothrombin time; PTT, partial thromboplastin time; SOFA, Sequential Organ Failure Assessment; SAPS II, Simplified Acute Physiology Score II; OASIS, Oxford Acute Severity of Illness Score; GCS, Glasgow Coma Scale; CCI, Charlson Comorbidity Index; CSF, cerebrospinal fluid; LOS, length of stay; ICU, intensive care unit.

**Table 2:** Associations of SHR with infection-related outcomes.

Categories	Suspected Infection		Culture-Positive Infection		Sepsis-3		Septic Shock	
	OR (95% CI)	*p*	OR (95% CI)	*p*	OR (95% CI)	*p*	OR (95% CI)	*p*
Model 1								
Continuous variable	1.12 (1.08, 1.15)	<0.001	1.09 (1.05, 1.13)	<0.001	1.14 (1.10, 1.18)	<0.001	1.15 (1.10, 1.19)	<0.001
Quartile								
Q1	Reference		Reference		Reference		Reference	
Q2	1.03 (0.75, 1.42)	0.850	1.24 (0.79, 1.96)	0.340	1.28 (0.88, 1.86)	0.200	1.88 (1.00, 3.53)	0.050
Q3	1.36 (0.99, 1.86)	0.060	1.56 (1.01, 2.43)	0.050	1.72 (1.19, 2.49)	0.004	2.71 (1.49, 4.92)	0.001
Q4	2.60 (1.90, 3.55)	<0.001	2.66 (1.76, 4.02)	<0.001	3.44 (2.42, 4.89)	<0.001	4.64 (2.64, 8.17)	<0.001
Model 2								
Continuous variable	1.10 (1.06, 1.14)	<0.001	1.08 (1.04, 1.12)	<0.001	1.09 (1.05, 1.13)	<0.001	1.11 (1.06, 1.16)	<0.001
Quartile								
Q1	Reference		Reference		Reference		Reference	
Q2	1.02 (0.74, 1.41)	0.900	1.26 (0.79, 1.99)	0.350	1.29 (0.88, 1.90)	0.190	1.91 (1.00, 3.64)	0.050
Q3	1.18 (0.85, 1.64)	0.320	1.38 (0.88, 2.17)	0.160	1.5 (1.03, 2.20)	0.040	2.22 (1.19, 4.14)	0.010
Q4	2.32 (1.68, 3.20)	<0.001	2.36 (1.55, 3.60)	<0.001	3.11 (2.16, 4.47)	<0.001	3.32 (1.85, 5.96)	<0.001
Model 3								
Continuous variable	1.08 (1.04, 1.12)	<0.001	1.05 (1.01, 1.09)	0.010	1.07 (1.03, 1.11)	<0.001	1.1 (1.05, 1.15)	<0.001
Quartile								
Q1	Reference		Reference		Reference		Reference	
Q2	1.07 (0.76, 1.50)	0.710	1.28 (0.80, 2.05)	0.310	1.39 (0.93, 2.07)	0.110	2.02 (1.03, 3.96)	0.040
Q3	1.13 (0.80, 1.60)	0.480	1.27 (0.79, 2.03)	0.320	1.54 (1.03, 2.29)	0.040	2.19 (1.14, 4.21)	0.010
Q4	1.98 (1.41, 2.78)	<0.001	1.87 (1.20, 2.91)	0.010	2.89 (1.98, 4.23)	<0.001	2.90 (1.56, 5.40)	<0.001

Continuous effect estimates are reported per 0.1-unit increase in SHR. OR, odds ratio; CI, confidence interval. Model 1 adjusted for age, sex, race, and HR. Model 2 additionally adjusted for CKD, HTN, AF, DM, CHF, HLP, AN, and procedure category. Model 3 additionally adjusted for RBC, platelet count, hemoglobin, hematocrit, bicarbonate, BUN, calcium, chloride, creatinine, sodium, potassium, INR, PT, PTT, GCS, and CCI.

**Table 3:** Incremental discrimination and reclassification after adding SHR to the clinical model.

	AUC (95% CI)	*p*	NRI (95% CI)	*p*	IDI (95% CI)	*p*
Suspected infection						
Clinical model	0.774 (0.750, 0.799)					
Clinical model + SHR	0.776 (0.751, 0.801)	0.337	0.087 (−0.016, 0.192)	0.108	0.004 (0.001, 0.007)	0.014
Culture-positive infection						
Clinical model	0.777 (0.746, 0.809)					
Clinical model + SHR	0.778 (0.746, 0.810)	0.401	0.035 (−0.106, 0.177)	0.621	0.000 (−0.001, 0.001)	0.798
Sepsis-3						
Clinical model	0.810 (0.786, 0.834)					
Clinical model + SHR	0.814 (0.790, 0.838)	0.067	0.172 (0.056, 0.290)	0.004	0.007 (0.002, 0.012)	0.002
Septic shock						
Clinical model	0.796 (0.761, 0.832)					
Clinical model + SHR	0.804 (0.769, 0.839)	0.077	0.206 (0.043, 0.372)	0.015	0.010 (0.002, 0.019)	0.018

The clinical model included age, sex, MBP, HTN, DM, AF, procedure category, CCI, RBC, WBC, platelet count, hemoglobin, hematocrit, bicarbonate, creatinine, INR, and GCS. AUC, area under the receiver operating characteristic curve; NRI, net reclassification improvement; IDI, integrated discrimination improvement; CI, confidence interval; SHR, stress hyperglycemia ratio.

**Table 4:** Associations of SHR with infection-related outcomes and 90-day mortality in nested clinical subsets.

	*n*	Events	Model 1		Model 2		Model 3	
			OR (95% CI)	*p*	OR (95% CI)	*p*	OR (95% CI)	*p*
SHR → suspected infection	1408	540	1.50 (1.33, 1.68)	<0.001	1.41 (1.25, 1.59)	<0.001	1.32 (1.16, 1.49)	<0.001
SHR → culture-positive infection (among suspected infection)	540	225	1.10 (0.94, 1.29)	0.220	1.08 (0.92, 1.26)	0.366	1.00 (0.84, 1.17)	0.958
SHR → Sepsis-3-defined sepsis (among suspected infection)	540	369	1.42 (1.18, 1.73)	<0.001	1.40 (1.16, 1.72)	<0.001	1.31 (1.07, 1.62)	0.012
SHR → septic shock (among Sepsis-3-defined sepsis)	369	148	1.31 (1.09, 1.58)	0.005	1.36 (1.12, 1.65)	0.002	1.36 (1.11, 1.68)	0.004
SHR → 90-day mortality (overall cohort)	1408	86	1.77 (1.49, 2.10)	<0.001	1.79 (1.50, 2.14)	<0.001	1.59 (1.29, 1.96)	<0.001
SHR → 90-day mortality (among Sepsis-3-defined sepsis)	369	54	1.45 (1.15, 1.83)	0.002	1.51 (1.19, 1.94)	<0.001	1.41 (1.06, 1.86)	0.016
SHR → 90-day mortality (among patients with septic shock)	148	27	1.38 (1.01, 1.90)	0.045	1.61 (1.13, 2.34)	0.009	1.63 (1.04, 2.66)	0.039

ORs are reported per 1-SD increase in SHR. Model 1 adjusted for age, sex, race, and HR. Model 2 additionally adjusted for CKD, HTN, AF, DM, CHF, HLP, AN, and procedure category. Model 3 additionally adjusted for RBC, platelet count, hemoglobin, hematocrit, bicarbonate, BUN, calcium, chloride, creatinine, sodium, potassium, INR, PT, PTT, GCS, and CCI. SHR, stress hyperglycemia ratio; SD, standard deviation; OR, odds ratio; CI, confidence interval; HR, heart rate; CKD, chronic kidney disease; HTN, hypertension; AF, atrial fibrillation; DM, diabetes mellitus; CHF, congestive heart failure; HLP, hyperlipidemia; AN, anemia; RBC, red blood cell count; BUN, blood urea nitrogen; INR, international normalized ratio; PT, prothrombin time; PTT, partial thromboplastin time; GCS, Glasgow Coma Scale; CCI, Charlson Comorbidity Index.

## Data Availability

The data analyzed in this study are publicly available in the MIMIC-IV database (version 2.2) at https://physionet.org/content/mimiciv/2.2/ (accessed on 11 August 2026) after completion of the required credentialing and data use agreement.

## Supplementary Materials

The following supporting information can be downloaded at https://www.mdpi.com/article/10.3390/jcm15197505/s1. Table S1: ICD-9-CM and ICD-10-PCS procedure codes used to identify eligible cranial neurosurgical procedures; Table S2: Baseline characteristics and clinical outcomes of patients included in versus excluded from the final analytic cohort; Table S3: Baseline characteristics and clinical outcomes of otherwise eligible patients with versus without available HbA1c measurements; Table S4: Baseline characteristics and clinical outcomes according to Sepsis-3 status; Table S5: Associations between SHR and infection-related outcomes in the 24-h lagged sensitivity analysis; Table S6: Exploratory model-based indirect associations between postoperative SHR and 90-day mortality; Figure S1: Dose–response relationships between SHR and infection-related outcomes stratified by glycemic status; Table S7: Associations between SHR and infection-related outcomes stratified by glycemic status; Figure S2: Associations between SHR quartiles and infection-related outcomes across glycemic-status groups.
