# Supplementary material for: Association of Postoperative Stress Hyperglycemia Ratio with Suspected Infection, Culture Positivity, Sepsis, and Septic Shock in 1408 Patients After Cranial Neurosurgery
Source: J Clin Med. 2026 Sep 27;15(19):7505. doi: 10.3390/jcm15197505 (PMC13665786; doi:10.3390/jcm15197505)
Supplement: Supplementary file 1 [file jcm-15-07505-s001.zip › jcm-4525883-supplementary.pdf]

Table S1. ICD-9-CM and ICD-10-PCS procedure codes used to identify eligible cranial neurosurgical procedures

Eligible procedure codes were mapped into the same six mutually exclusive analytic categories used in the main manuscript and Table 1: cranial resection/biopsy, cerebrovascular procedure, intracranial evacuation/drainage, CSF diversion/drainage, skull-base/pituitary/cranial nerve procedure, and other cranial procedure.

ICD-9	9-0159 9-0131 9-0212 9-0221 9-0151 9-3951 9-022 9-0234 9-0110 9-0239 9-0124 9-0206 9-0125 9-3812 9-0202 9-0114 9-0153 9-0123 9-0204 9-0242 9-0243 9-0109 9-0401 9-0128 9-0211 9-0222 9-0449 9-0441 9-0479 9-0127 9-016 9-0761 9-3928 9-0404 9-0407 9-0126 9-0412 9-046 9-0442 9-045 9-3802 9-0112 9-0403 9-0132 9-0233 9-0241 9-0764 9-3861 9-0152 9-0214 9-0292 9-0299 9-0371 9-0474 9-1753 9-1754 9-3801 9-3831 9-3835
ICD-10	009630Z 00B70ZZ 00U20KZ 00C40ZZ 00B10ZZ 03LG0CZ 03VG0CZ 00B00ZZ 00N00ZZ 00163J6 00B70ZX 00NW0ZZ 00BC0ZZ 00U207Z 009400Z 0GB04ZZ 00940ZZ 009430Z 00C43ZZ 05BL0ZZ 4A103BD 00U20JZ 00943ZZ 03BG0ZZ 00B00ZX 00P6X0Z 03CK0ZZ 03CL0ZZ 00B20ZZ 00C00ZZ 00C70ZZ 009600Z 00C30ZZ 0W310ZZ 00H032Z 00B73ZX 00NC0ZZ 00160J6 0GB00ZZ 03CM0ZZ 00B60ZZ 0NB00ZZ 00BN0ZZ 00QT0ZZ 01N10ZZ 0W360ZZ 00B03ZX 00B10ZX 00CC0ZZ 03CL3ZZ 00164J6 03UL0KZ 03CH0ZZ 00B20ZX 00NK0ZZ 00UT0JZ 03CN0ZZ 03UK0KZ 0NP004Z 00BC0ZX 00Q20ZZ 00P60JZ 00P630Z 00BM0ZZ 037G3ZZ 03SS0ZZ 3E0Q3GC 00970ZZ 03CJ0ZZ 0NU007Z 00U10KZ 00W63JZ 037J3DZ 03VN3DZ 0NR007Z 0NU00JZ 009300Z 00B93ZX 00BW0ZZ 05BM0ZZ 05BN0ZZ 09UX47Z 00900ZZ 00K70ZZ 00NM0ZZ 03UJ0KZ 0GB04ZX 0GT04ZZ 00BK0ZZ 00D20ZZ 00U247Z 0W21X0Z 8C01X6J 00930ZZ 00BN0ZX 00C20ZZ 00H002Z 00P0X2Z 00U24KZ 00UT07Z 031J0JY 031S0ZG 0NR00KZ 009640Z 00B03ZZ 00B14ZZ 00CW0ZZ 00J00ZZ 0W9100Z 0WH133Z 0020X0Z 008Q4ZZ 00B04ZZ 00B23ZX 00B64ZZ 00B74ZZ 00BQ0ZZ 00C50ZZ 00D10ZZ 00N10ZZ 00P00JZ 00R20JZ 00U147Z 00U14KZ 0W300ZZ 0W910ZZ

Table S2. Baseline characteristics and clinical outcomes of patients included in versus excluded from the final analytic cohort.

Variables	Included (n = 1,408)	Excluded (n = 3,693)	P
Demographics			
Age, years	62.56 ± 15.71	59.56 ± 17.23	<0.001
Sex, female, n (%)	687 (48.79)	1745 (47.25)	0.340
Weight, kg	82.17 ± 21.40	80.35 ± 28.43	0.290
Race			<0.001
Other, n (%)	633 (44.96)	1417 (38.37)	
White, n (%)	775 (55.04)	2276 (61.63)	
Vital signs			
HR mean, beats/min	79.97 ± 13.69	79.75 ± 13.75	0.610
SBP mean, mmHg	127.54 ± 13.11	125.54 ± 12.46	<0.001
DBP mean, mmHg	66.57 ± 10.20	65.96 ± 9.67	0.050
MBP mean, mmHg	85.21 ± 9.41	84.26 ± 8.95	<0.010
Comorbidity			
CKD, n (%)	118 (8.38)	223 (6.04)	<0.010
HTN, n (%)	736 (52.27)	1628 (44.08)	<0.001
AF, n (%)	147 (10.44)	294 (7.96)	<0.010
DM, n (%)	377 (26.78)	546 (14.78)	<0.001
CHF, n (%)	110 (7.81)	206 (5.58)	<0.010
HLP, n (%)	601 (42.68)	1169 (31.65)	<0.001
Laboratory indicators			
RBC (10^12/L)	4.45 ± 0.61	4.35 ± 0.65	<0.001
WBC (10^9/L)	9.37 ± 4.32	10.43 ± 8.66	<0.001
Platelet (10^9/L)	241.13 ± 75.26	246.71 ± 89.08	0.030
Hemoglobin (g/dL)	13.36 ± 1.77	13.11 ± 1.94	<0.001
Hematocrit (%)	37.45 ± 5.20	36.99 ± 5.38	<0.010

BUN (mg/dL)	17.03 ± 9.42	17.07 ± 10.04	0.910
Calcium (mg/dL)	8.74 ± 0.61	8.73 ± 0.67	0.560
Chloride (mmol/L)	106.33 ± 5.21	106.39 ± 5.35	0.680
Creatinine (mg/dL)	1.00 ± 0.89	0.95 ± 0.66	0.060
Lactate (mmol/L)	1.96 ± 1.44	2.00 ± 1.46	0.320
Sodium (mmol/L)	140.99 ± 4.51	140.91 ± 4.36	0.570
Potassium (mmol/L)	4.20 ± 0.67	4.21 ± 0.64	0.670
TG (mg/dL)	155.33 ± 112.00	160.03 ± 137.50	0.390
Glucose (mg/dL)	134.53 ± 59.67	126.02 ± 49.22	<0.001
INR	1.27 ± 0.82	1.25 ± 0.64	0.370
PT (seconds)	13.97 ± 8.26	13.77 ± 7.72	0.440
PTT (seconds)	33.32 ± 19.15	30.82 ± 16.05	<0.001
Disease severity score			
SOFA	2.62 ± 2.19	2.55 ± 2.33	0.330
SAPS II	29.34 ± 11.07	30.19 ± 11.78	0.020
OASIS	27.35 ± 8.18	27.22 ± 8.38	0.590
GCS	13.22 ± 2.70	13.31 ± 2.71	0.300
CCI	4.13 ± 2.52	4.19 ± 3.15	0.510
Outcomes			
ICU death, n (%)	80 (5.68)	217 (5.88)	0.840
In-hospital death, n (%)	96 (6.82)	298 (8.07)	0.150
30-day mortality, n (%)	84 (5.97)	217 (5.88)	0.960
90-day mortality, n (%)	86 (6.11)	224 (6.07)	1.000

HR, heart rate; SBP, systolic blood pressure; DBP, diastolic blood pressure; MBP, mean blood pressure; CKD, chronic kidney disease; HTN, hypertension; AF, atrial fibrillation; DM, diabetes mellitus; CHF, congestive heart failure; HLP, hyperlipidemia; TG, triglycerides; INR, international normalized ratio; PT, prothrombin time; PTT, partial thromboplastin time. SOFA, sequential organ failure assessment; SAPS II, Simplified Acute Physiology Score II; OASIS, Oxford Acute Severity of Illness Score; GCS, Glasgow Coma Scale; CCI, Charlson Comorbidity Index.

Table S3. Baseline characteristics and clinical outcomes of otherwise eligible patients with versus without available HbA1c measurements.

Variables	HbA1c available (n = 1,408)	HbA1c unavailable (n = 1,043)	P
Demographics			
Age, years	62.56 ± 15.71	58.68 ± 17.63	<0.001
Sex, female, n (%)	687 (48.79)	515 (49.38)	0.340
Weight, kg	82.17 ± 21.40	77.53 ± 18.37	<0.001
Race			<0.001
Other, n (%)	633 (44.96)	382 (36.63)	
White, n (%)	775 (55.04)	661 (63.37)	
Vital signs			
HR mean, beats/min	79.97 ± 13.69	79.59 ± 13.61	0.490
SBP mean, mmHg	127.54 ± 13.11	125.17 ± 12.17	<0.001
DBP mean, mmHg	66.57 ± 10.20	65.56 ± 9.41	0.010
MBP mean, mmHg	85.21 ± 9.41	83.88 ± 8.68	<0.010
Comorbidity			
CKD, n (%)	118 (8.38)	50 (4.79)	<0.001
HTN, n (%)	736 (52.27)	416 (39.88)	<0.001
AF, n (%)	147 (10.44)	69 (6.62)	<0.010
DM, n (%)	377 (26.78)	113 (10.83)	<0.001
CHF, n (%)	110 (7.81)	39 (3.74)	<0.001
HLP, n (%)	601 (42.68)	278 (26.65)	<0.001
Laboratory indicators			
RBC (10^12/L)	4.45 ± 0.61	4.38 ± 0.62	<0.01
WBC (10^9/L)	9.37 ± 4.32	10.56 ± 7.84	<0.001
Platelet (10^9/L)	241.13 ± 75.26	244.56 ± 86.24	0.310
Hemoglobin (g/dL)	13.36 ± 1.77	13.16 ± 1.87	<0.010
Hematocrit (%)	37.45 ± 5.20	37.16 ± 5.18	0.190

BUN (mg/dL)	17.03 ± 9.42	16.52 ± 9.83	0.190
Calcium (mg/dL)	8.74 ± 0.61	8.70 ± 0.65	0.130
Chloride (mmol/L)	106.33 ± 5.21	106.67 ± 5.24	0.110
Creatinine (mg/dL)	1.00 ± 0.89	0.91 ± 0.52	<0.010
Lactate (mmol/L)	1.96 ± 1.44	2.07 ± 1.59	0.120
Sodium (mmol/L)	140.99 ± 4.51	141.09 ± 4.45	0.600
Potassium (mmol/L)	4.20 ± 0.67	4.16 ± 0.64	0.200
TG (mg/dL)	155.33 ± 112.00	164.01 ± 157.25	0.550
Glucose (mg/dL)	134.53 ± 59.67	124.99 ± 49.12	<0.001
INR	1.27 ± 0.82	1.23 ± 0.63	0.180
PT (seconds)	13.97 ± 8.26	13.59 ± 7.78	0.260
PTT (seconds)	33.32 ± 19.15	30.16 ± 15.52	<0.001
Disease severity score			
SOFA	2.62 ± 2.19	2.46 ± 2.43	0.090
SAPS II	29.34 ± 11.07	29.24 ± 12.23	0.840
OASIS	27.35 ± 8.18	26.98 ± 8.57	0.280
GCS	13.22 ± 2.70	13.41 ± 2.60	0.080
CCI	4.13 ± 2.52	3.76 ± 3.01	<0.010
Outcomes			
ICU death, n (%)	80 (5.68)	74 (7.09)	0.180
In-hospital death, n (%)	96 (6.82)	94 (9.01)	0.050
30-day mortality, n (%)	84 (5.97)	74 (7.09)	0.300
90-day mortality, n (%)	86 (6.11)	76 (7.29)	0.280

HR, heart rate; SBP, systolic blood pressure; DBP, diastolic blood pressure; MBP, mean blood pressure; CKD, chronic kidney disease; HTN, hypertension; AF, atrial fibrillation; DM, diabetes mellitus; CHF, congestive heart failure; HLP, hyperlipidemia; TG, triglycerides; INR, international normalized ratio; PT, prothrombin time; PTT, partial thromboplastin time. SOFA, sequential organ failure assessment; SAPSII, simplified acute physiology score; OASIS, oxford acute severity of illness score; GCS, Glasgow coma scale; CCI, Charlson Comorbidity Index.

Table S4. Baseline characteristics and clinical outcomes according to Sepsis-3 status.

Variables	Total (n = 1,408)	No Sepsis-3 (n = 1,039)	Sepsis-3 (n = 369)	P
Demographics				
Age, years	62.56 ± 15.71	62.20 ± 15.72	63.58 ± 15.65	0.150
Sex, female, n (%)	687 (48.79)	514 (49.47)	173 (46.88)	0.430
Weight, kg	82.17 ± 21.40	82.20 ± 21.34	82.09 ± 21.59	0.930
Race				<0.001
Other, n (%)	633 (44.96)	431 (41.48)	202 (54.74)	
White, n (%)	775 (55.04)	608 (58.52)	167 (45.26)	
Vital signs				
HR mean, beats/min	79.97 ± 13.69	79.04 ± 12.71	82.59 ± 15.86	<0.001
SBP mean, mmHg	127.54 ± 13.11	127.27 ± 12.63	128.28 ± 14.37	0.230
DBP mean, mmHg	66.57 ± 10.20	67.15 ± 10.07	64.95 ± 10.40	<0.001
MBP mean, mmHg	85.21 ± 9.41	85.74 ± 9.31	83.72 ± 9.55	<0.001
Comorbidity				
CKD, n (%)	118 (8.38)	84 (8.08)	34 (9.21)	0.570
HTN, n (%)	736 (52.27)	528 (50.82)	208 (56.37)	0.080
AF, n (%)	147 (10.44)	94 (9.05)	53 (14.36)	<0.01
DM, n (%)	377 (26.78)	266 (25.60)	111 (30.08)	0.110
CHF, n (%)	110 (7.81)	58 (5.58)	52 (14.09)	<0.001
HLP, n (%)	601 (42.68)	449 (43.21)	152 (41.19)	0.540
AN, n (%)	221 (15.70)	124 (11.93)	97 (26.29)	<0.001
AKI, n (%)	101 (7.17)	45 (4.33)	56 (15.18)	<0.001
Laboratory indicators				
RBC (10^12/L)	4.45 ± 0.61	4.48 ± 0.59	4.37 ± 0.68	<0.01
WBC (10^9/L)	9.37 ± 4.32	8.88 ± 3.95	10.77 ± 4.96	<0.001
Platelet (10^9/L)	241.13 ± 75.26	246.61 ± 74.10	225.73 ± 76.45	<0.001

Hemoglobin (g/dL)	13.36 ± 1.77	13.46 ± 1.68	13.06 ± 1.97	<0.001
Hematocrit (%)	37.45 ± 5.20	37.13 ± 4.99	38.34 ± 5.69	<0.001
HbA1c (%)	6.24 ± 1.33	6.20 ± 1.24	6.33 ± 1.56	0.140
Monocytes (%)	6.33 ± 2.70	6.43 ± 2.65	6.06 ± 2.83	0.030
Lymphocytes (%)	20.67 ± 11.57	22.12 ± 11.31	16.60 ± 11.33	<0.001
Anion gap (mmol/L)	15.06 ± 3.51	14.70 ± 3.38	16.09 ± 3.67	<0.001
Bicarbonate (mmol/L)	23.60 ± 2.96	23.50 ± 2.83	23.88 ± 3.28	0.050
BUN (mg/dl)	17.03 ± 9.42	16.16 ± 8.47	19.50 ± 11.36	<0.001
Calcium (mg/dL)	8.74 ± 0.61	8.72 ± 0.58	8.79 ± 0.67	0.110
Chloride (mmol/L)	106.33 ± 5.21	105.98 ± 4.68	107.30 ± 6.39	<0.001
Creatinine (mg/dL)	1.00 ± 0.89	0.96 ± 0.90	1.12 ± 0.84	<0.01
Lactate (mmol/L)	1.96 ± 1.44	1.86 ± 1.41	2.22 ± 1.47	<0.001
Sodium (mmol/L)	140.99 ± 4.51	140.59 ± 3.88	142.12 ± 5.81	<0.001
Potassium (mmol/L)	4.20 ± 0.67	4.13 ± 0.57	4.38 ± 0.87	<0.001
TG (mg/dL)	155.33 ± 112.00	154.27 ± 117.38	158.31 ± 95.32	0.510
Glucose (mg/dL)	134.53 ± 59.67	128.05 ± 55.96	152.80 ± 65.78	<0.001
INR	1.27 ± 0.82	1.22 ± 0.41	1.42 ± 1.44	<0.01
PT (seconds)	13.97 ± 8.26	13.52 ± 5.75	15.25 ± 12.85	0.010
PTT (seconds)	33.32 ± 19.15	32.72 ± 18.62	34.99 ± 20.52	0.060
Disease severity score				
SOFA	2.62 ± 2.19	2.17 ± 1.85	3.88 ± 2.57	<0.001
SAPSII	29.34 ± 11.07	27.34 ± 10.12	34.95 ± 11.71	<0.001
OASIS	27.35 ± 8.18	25.31 ± 7.45	33.11 ± 7.38	<0.001
GCS	13.22 ± 2.70	13.54 ± 2.29	12.34 ± 3.46	<0.001
CCI	4.13 ± 2.52	3.96 ± 2.49	4.63 ± 2.56	<0.001
Treatment				
Ventilation, n (%)	441 (31.32)	198 (19.06)	243 (65.85)	<0.001
Vasoactive drug, n (%)	214 (15.20)	84 (8.08)	130 (35.23)	<0.001
Procedure category				<0.001

Cerebrovascular procedure	304 (21.59)	266 (25.60)	38 (10.30)	
Cranial resection/biopsy	383 (27.20)	341 (32.82)	42 (11.38)	
CSF diversion/drainage	186 (13.21)	83 (7.99)	103 (27.91)	
Intracranial evacuation/drainage	146 (10.37)	92 (8.85)	54 (14.63)	
Other cranial procedure	244 (17.33)	150 (14.44)	94 (25.47)	
Skull-base/pituitary/cranial nerve procedure	145 (10.30)	107 (10.30)	38 (10.30)	
Length of stay (LOS)				
LOS in hospital	11.24 ± 11.21	7.78 ± 6.70	20.98 ± 14.98	<0.001
LOS in ICU	5.71 ± 6.93	3.36 ± 3.40	12.30 ± 9.59	<0.001
Outcomes				
ICU death, n (%)	80 (5.68)	29 (2.79)	51 (13.82)	<0.001
In-hospital death, n (%)	96 (6.82)	37 (3.56)	59 (15.99)	<0.001
30-day mortality, n (%)	84 (5.97)	32 (3.08)	52 (14.09)	<0.001
90-day mortality, n (%)	86 (6.11)	32 (3.08)	54 (14.63)	<0.001

SHR, stress hyperglycemia ratio; HR, heart rate; SBP, systolic blood pressure; DBP, diastolic blood pressure; MBP, mean blood pressure; CKD, chronic kidney disease; HTN, hypertension; AF, atrial fibrillation; DM, diabetes mellitus; CHF, congestive heart failure; HLP, hyperlipidemia; AN, anemia; AKI, acute kidney injury; RBC, red blood cell count; WBC, white blood cell count; HbA1c, glycated hemoglobin; BUN, blood urea nitrogen; TG, triglycerides; INR, international normalized ratio; PT, prothrombin time; PTT, partial thromboplastin time; SOFA, Sequential Organ Failure Assessment; SAPS II, Simplified Acute Physiology Score II; OASIS, Oxford Acute Severity of Illness Score; GCS, Glasgow Coma Scale; CCI, Charlson Comorbidity Index; CSF, cerebrospinal fluid; LOS, length of stay; ICU, intensive care unit.

Table S5. Associations between stress hyperglycemia ratio and infection-related outcomes in the 24-hour lagged sensitivity analysis.

Categories	Suspected infection		Culture-positive infection		Sepsis-3		Septic shock	
	OR (95% CI)	P	OR (95% CI)	P	OR (95% CI)	P	OR (95% CI)	P
Model 1								
Continuous variable	1.11 (1.07, 1.15)	<0.001	1.09 (1.05, 1.13)	<0.001	1.14 (1.10, 1.18)	<0.001	1.15 (1.10, 1.20)	<0.001
Quartile								
Q1	Reference		Reference		Reference		Reference	
Q2	1.02 (0.73, 1.42)	0.900	1.29 (0.79, 2.12)	0.300	1.25 (0.83, 1.88)	0.280	1.71 (0.85, 3.44)	0.140
Q3	1.30 (0.93, 1.80)	0.130	1.56 (0.96, 2.53)	0.070	1.70 (1.14, 2.54)	0.010	2.43 (1.25, 4.73)	0.010
Q4	2.44 (1.76, 3.38)	<0.001	2.65 (1.69, 4.16)	<0.001	3.29 (2.25, 4.81)	<0.001	4.34 (2.32, 8.11)	<0.001
Model 2								
Continuous variable	1.09 (1.06, 1.13)	<0.001	1.07 (1.03, 1.12)	0.001	1.12 (1.08, 1.16)	<0.001	1.14 (1.09, 1.19)	<0.001
Quartile								
Q1	Reference		Reference		Reference		Reference	
Q2	1.01 (0.72, 1.41)	0.960	1.29 (0.79, 2.13)	0.340	1.30 (0.83, 2.01)	0.250	1.65 (0.81, 3.35)	0.070
Q3	1.13 (0.80, 1.59)	0.500	1.38 (0.84, 2.27)	0.180	1.28 (0.83, 1.98)	0.260	2.08 (1.05, 4.11)	0.050
Q4	2.22 (1.58, 3.10)	<0.001	2.40 (1.51, 3.81)	<0.001	2.27 (1.50, 3.44)	<0.001	3.79 (2.01, 7.13)	<0.001
Model 3								
Continuous variable	1.08 (1.04, 1.12)	<0.001	1.05 (1.01, 1.10)	0.01	1.10 (1.06, 1.15)	<0.001	1.13 (1.07, 1.19)	<0.001
Quartile								
Q1	Reference		Reference		Reference		Reference	
Q2	1.05 (0.74, 1.49)	0.780	1.34 (0.81, 2.22)	0.260	1.39 (0.88, 2.20)	0.160	1.75 (0.84, 3.68)	0.060
Q3	1.08 (0.76, 1.55)	0.660	1.41 (0.85, 2.33)	0.180	1.25 (0.79, 1.99)	0.340	2.01 (0.98, 4.12)	0.080
Q4	1.90 (1.33, 2.71)	<0.001	2.20 (1.37, 3.53)	0.001	1.92 (1.23, 3.00)	0.004	3.91 (1.86, 8.25)	<0.001

In the 24-hour lagged sensitivity analysis, patients who developed the corresponding outcome within 24 hours after SHR assessment were excluded, and only incident outcomes occurring more than 24 hours after SHR assessment were included. OR, odds ratio; CI, confidence interval. Model 1 adjusted for age, sex, race, and HR. Model 2 additionally adjusted for CKD, HTN, AF, DM, CHF, HLP, AN, and procedure category. Model 3 additionally adjusted for RBC, platelet count, hemoglobin, hematocrit, bicarbonate, BUN, calcium, chloride, creatinine, sodium, potassium, INR, PT, PTT, GCS, and CCI.

Table S6. Exploratory model-based indirect associations between postoperative SHR and 90-day mortality.

Analysis	n	Mediator events	Outcome events	ACME (95% CI)	P	ADE (95% CI)	P	Total effect (95% CI)	P	Estimated proportion mediated (95% CI)
SHR → Sepsis-3 → 90-day mortality	1,408	369	86	0.0070 (0.0029, 0.0138)	<0.001	0.0287 (0.0180, 0.0427)	<0.001	0.0357 (0.0241, 0.0507)	<0.001	19.7% (8.6%, 37.8%)
SHR → septic shock → 90-day mortality	1,408	148	86	0.0054 (0.0010, 0.0094)	0.006	0.0308 (0.0193, 0.0452)	<0.001	0.0361 (0.0232, 0.0490)	<0.001	14.8% (2.9%, 28.0%)

ACME, average mediation effect; ADE, average direct effect; CI, confidence interval; SHR, stress hyperglycemia ratio. SHR was standardized before analysis. Effect estimates represent the change associated with an increase in SHR from the population mean to 1 standard deviation above the mean. Confidence intervals were estimated using 5,000 nonparametric bootstrap resamples. Because the assumptions required for causal mediation cannot be fully verified in this retrospective observational cohort, these analyses should be interpreted as exploratory model-based indirect associations rather than evidence of causal mediation.

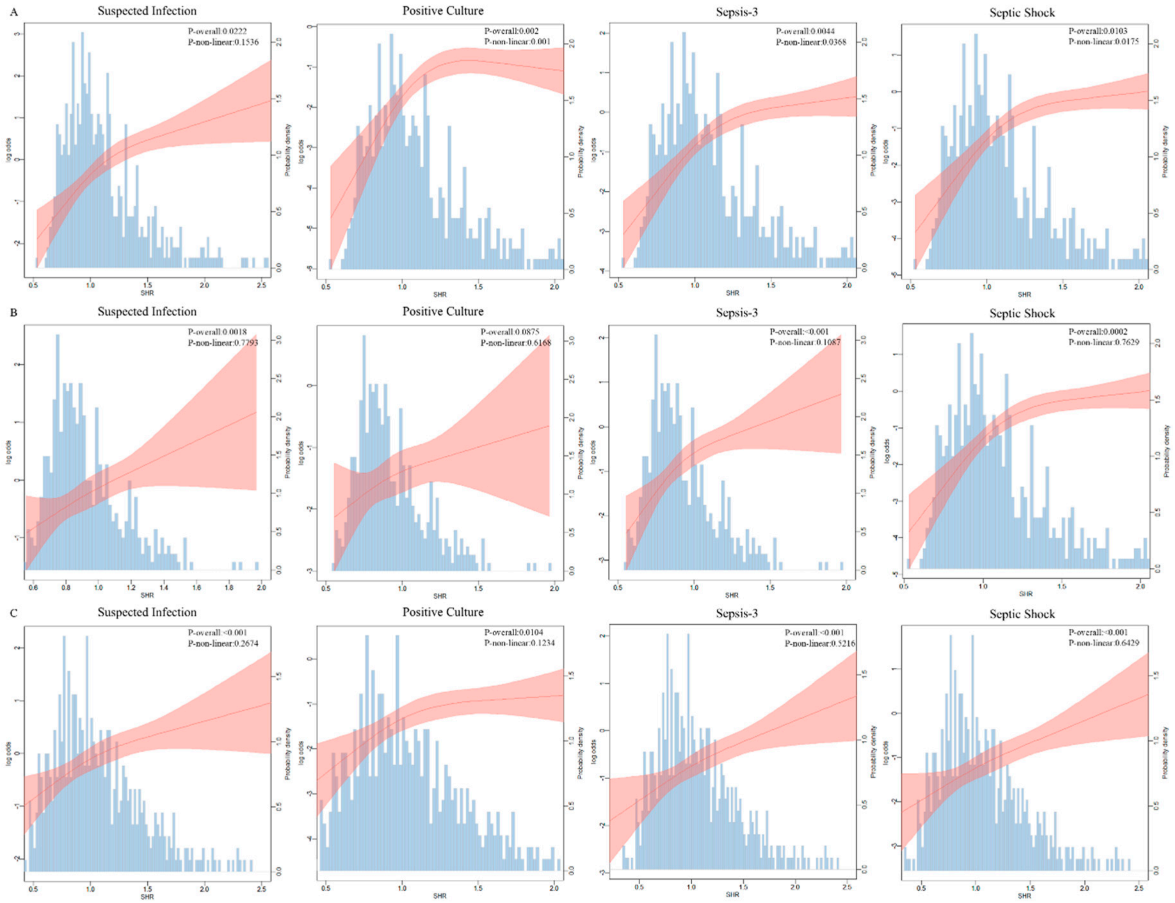

Figure S1. Dose–response relationships between SHR index and infection-related outcomes in patients after cranial neurosurgical procedures stratified by glucose metabolic states (A: NGR; B: Pre-DM; C: DM).

Table S7. Associations between SHR and infection-related outcomes stratified by glycemic status.

Categories	Suspected infection		Culture-positive infection		Sepsis-3		Septic shock	
	OR (95% CI)	P	OR (95% CI)	P	OR (95% CI)	P	OR (95% CI)	P
Patients with NGR								
Continuous variable	1.11 (1.05, 1.18)	<0.001	1.08 (1.01, 1.15)	0.020	1.11 (1.05, 1.18)	<0.001	1.15 (1.07, 1.23)	<0.001
Quartile								
Q1	Reference		Reference		Reference		Reference	
Q2	0.89 (0.47, 1.68)	0.710	1.26 (0.36, 4.44)	0.720	1.60 (0.63, 4.08)	0.330	2.82 (0.77, 10.29)	0.120
Q3	1.64 (0.89, 3.02)	0.110	3.06 (0.98, 9.61)	0.050	2.66 (1.10, 6.43)	0.030	5.70 (1.68, 19.33)	0.010
Q4	2.49 (1.31, 4.74)	0.010	4.16 (1.33, 12.98)	0.010	3.28 (1.35, 7.96)	0.010	4.96 (1.47, 16.74)	0.010
Patients with Pre-DM								
Continuous variable	1.14 (1.04, 1.25)	0.004	1.10 (1.00, 1.21)	0.060	1.25 (1.14, 1.38)	<0.001	1.26 (1.12, 1.42)	<0.001
Quartile								
Q1	Reference		Reference		Reference		Reference	
Q2	1.24 (0.78, 1.98)	0.350	1.56 (0.80, 3.03)	0.190	1.76 (0.97, 3.22)	0.070	1.35 (0.72, 2.54)	0.350
Q3	2.06 (1.24, 3.40)	0.005	1.61 (0.80, 3.24)	0.190	2.83 (1.51, 5.29)	0.001	2.40 (1.25, 4.60)	0.010
Q4	2.18 (1.18, 4.00)	0.010	1.74 (0.77, 3.92)	0.180	3.61 (1.76, 7.40)	<0.001	3.10 (1.49, 6.43)	0.002
Patients with DM								
Continuous variable	1.08 (1.03, 1.14)	0.003	1.06 (1.00, 1.13)	0.040	1.10 (1.04, 1.16)	<0.001	1.09 (1.02, 1.17)	0.010
Quartile								
Q1	Reference		Reference		Reference		Reference	
Q2	1.57 (0.85, 2.90)	0.150	2.42 (1.09, 5.36)	0.030	1.46 (0.73, 2.93)	0.290	2.17 (0.99, 4.76)	0.050
Q3	1.47 (0.81, 2.68)	0.210	1.54 (0.70, 3.37)	0.280	1.45 (0.75, 2.83)	0.270	1.47 (0.69, 3.17)	0.320
Q4	2.14 (1.23, 3.73)	0.010	2.84 (1.41, 5.74)	0.004	2.13 (1.17, 3.89)	0.010	2.96 (1.49, 5.88)	0.002

Continuous effect estimates are reported per 0.1-unit increase in SHR. OR, odds ratio; CI, confidence interval; NGR, normal glucose regulation; Pre-DM, prediabetes; DM, diabetes mellitus. Models were adjusted for age, sex, race, HR, CKD, HTN, AF, CHF, HLP, AN, procedure category, RBC, platelet count, hemoglobin, hematocrit, bicarbonate, BUN, calcium, chloride, creatinine, sodium, potassium, INR, PT, PTT, GCS, and CCI.

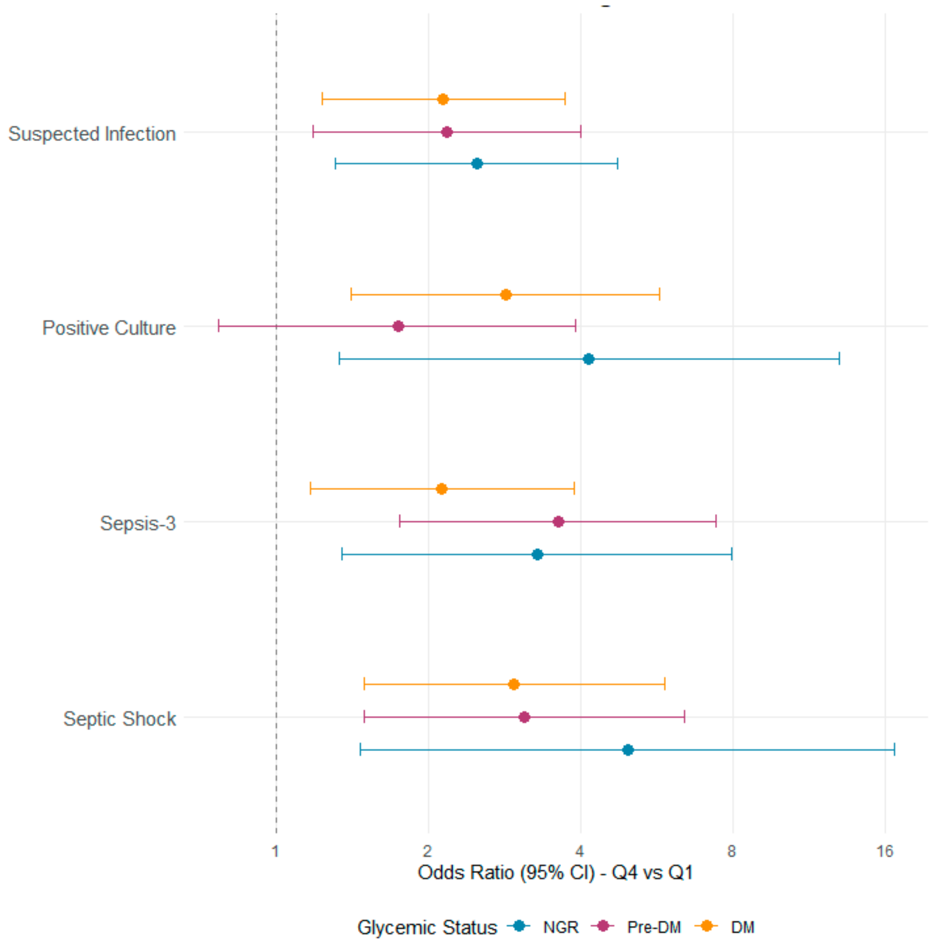

Figure S2. Association between SHR index quartiles and infection outcomes across different glycemic status groups.
